# Development of the clinical learning evaluation questionnaire for undergraduate clinical education: factor structure, validity, and reliability study

**DOI:** 10.1186/1472-6920-14-44

**Published:** 2014-03-04

**Authors:** Ali I AlHaqwi, Jeroen Kuntze, Henk T van der Molen

**Affiliations:** 1 Department of Family Medicine, King Saud Ben Abdul-Aziz University for Health Sciences, PO Box: 69416, Riyadh 11547, Saudi Arabia; 2 Faculty of Psychology and Educational Sciences, Open University of the Netherlands, Heerlen, The Netherlands; 3 Department of Psychology, Faculty of Social Sciences, Erasmus University Rotterdam, Rotterdam, The Netherlands

## Abstract

**Background:**

Teaching and learning of clinical skills for undergraduate medical students usually takes place during the clinical clerkship. Therefore, it is of vital importance to ensure the effectiveness of the rotations within this clerkship. The aims of this study were to develop an instrument that measures the effectiveness of the clinical learning environment, to determine its factor structure, and to find first evidence for the reliability and validity of the total scale and the different factors.

**Methods:**

The Clinical Learning Evaluation Questionnaire (CLEQ) is an instrument, consisting of 40 items, which have been developed after consideration of the results of a qualitative study that investigated the important factors influencing clinical learning, both from the perspective of students, as well as teachers. Results of relevant literature that investigated this issue were also incorporated in the CLEQ. This instrument was administered to a sample of students (N = 182) from three medical colleges in Riyadh city, the capital of Saudi Arabia. The factor structure of the CLEQ (Principal component analysis, Oblimin rotation) and reliability of the factor scales (Cronbach’s α) were determined. Hypotheses concerning the correlations between the different factors were tested to investigate their convergent and divergent validity.

**Results:**

One hundred and nine questionnaires were returned. The factor analysis yielded six factors: F1 Cases (8 items), F2 Authenticity of clinical experience (8 items), F3 Supervision (8 items), F4 Organization of the doctor-patient encounter (4 items), F5 Motivation to learn (5 items), and F6 Self awareness (4 items). The overall internal consistency (α) of the CLEQ was 0.88, and the reliabilities (Cronbach’s α) of the six factors varied from .60 to .86. Hypotheses concerning the correlations between the different factors were partly confirmed, which supported the convergent validity of the factors, but not their divergent validity. Significant differences were found between the scores of the students of the three different schools on the factors Supervision and Organization of patient-doctor encounter.

**Conclusions:**

The results of this study demonstrated that CLEQ is a multidimensional and reliable instrument. It can be utilized as an evaluation tool for clinical teaching activities, both by educators as well as students. Further research is needed into the validity of the CLEQ.

## Background

The essence of medical education is to graduate competent medical professionals, who have the essential clinical skills required for the management of common medical problems. As the process of clinical training takes place mainly during the clinical rotations, it is of vital importance to ensure that medical students are exposed adequately and early to clinical situations during their training.

Learning in the clinical setting is a complex process and could be influenced by many factors, such as the quality of the supervision, exposure to a variety of clinical experiences, quality of feedback and the length of time spent with patients [1-7]. The impact of these factors on the clinical learning of undergraduate medical students is variable [2]. However, students’ performance on clinical examinations was found to be positively associated with exposure to a large variety of clinical cases and the provision of feedback from the supervisors [3]. Interestingly, the perception of medical students showed as well, that these factors are important for the enhancement of their clinical learning [8].

There are many tools to measure the educational environments in general, in different settings and different disciplines. Among them are: the Dundee Ready Education Environment Measure (DREEM), the Postgraduate Hospital Educational Environment Measure (PHEEM) and The Clinical Learning Environment Inventory (CLEI) [9-11]. These instruments aim to explore the educational environment in general and its effect on the learning process. Factors related to academic atmosphere, facilities, and psychosocial characteristics of the clinical learning environment were the main focus of these instruments.

Despite the increasing interest to measure the effectiveness of the clinical rotations for undergraduate medical students, only a few studies have addressed the quality of teaching in undergraduate clinical education [12-15]. The study by Pololi and Price was one of the first to propose a measurement of the effectiveness of the clinical learning environment and the learning process of undergraduate medical students [12]. Another instrument is the Cleveland Clinical Teaching Effectiveness Inventory (CCTEI), which has been developed to evaluate the quality of the teaching process. This instrument has been tested and validated for the measurement of effectiveness of the teaching process for undergraduate and postgraduate medical students, both at an individual and at a group level [13,14]. These two instruments focused on major issues that influence students’ learning, such as teacher-learner relationship, self efficacy, and physician-patient relationship. However, the features of the clinical experiences and the organizational issues were not explored.

The relationship between different variables involved in the clinical learning process of the undergraduate medical students have been studied and a model that explains how these variables work together for the effectiveness of the clinical rotation was proposed by Dolmans, et al. [16]. This model investigated the influence of factors related to patient-mix, supervision and organizational issues in the effectiveness of clinical rotation. However, other important factors, such as motivation of students and features of the clinical experiences as authenticity that could influence this process as well were not investigated.

Based on the available data and considering possible factors that could influence the process of clinical learning, the present study was carried out to develop and test an instrument that could evaluate the quality of clinical education of undergraduate medical students better than the instruments mentioned above.

The first aim of this study was to investigate the reliability and factor structure of the instrument, that we have developed, and that we have called the Clinical Learning Evaluation Questionnaire (CLEQ). It was intended to measure five factors that were found in our previous study (8) and that are often mentioned in the literature, namely: (1) Provision of clinical cases, (2) Authenticity of clinical experiences, (3) Quality of Supervision (further to be referred to as Supervision), (4) Organization of the doctor-patient encounter, and (5) Motivation to learn.

The second aim of the study was to investigate the convergent and discriminant validity of the factor scales by testing a number of hypotheses concerning the question, how the factors that we have found are correlating. The third aim was to investigate whether there are differences between the three schools on the different factors, which would imply that the CLEQ is able to discriminate between the effectiveness of different clinical learning environments. The following hypotheses concerning the correlations between the five factors were formulated.

Hypothesis 1: Factor 1 Cases, which measures students’ satisfaction with the number and variety of cases seen during clinical rotation, is expected to correlate positively with factor 2 Authenticity of the clinical learning experiences and factor 5 Motivation.

Hypothesis 2: Factor 2 Authenticity of the clinical experiences was also expected to correlate with factor 5 Motivation. We assumed that the more authentic the learning experiences are, the more motivation will be developed.

Hypothesis 3: Factor 3 Supervision will be positively correlated with factor 4 Organization of the doctor-patient encounter and factor 5 Motivation. No or no significant correlations were expected between Factor 3 and Factor 1 Cases and Factor 2 Authenticity of the clinical learning experiences.

Hypothesis 4: Factor 4 Organization of the doctor-patient encounter will be related to factor 5 Motivation. The students’ view to the organizational issues of the clinical rotations is not expected to correlate positively with their opinion regarding issues of the clinical cases (factor 1), authenticity of the clinical experiences (factor 2) and supervision (factor 3).

## Methods

### Development of the Clinical Learning Evaluation Questionnaire (CLEQ)

The first version of the CLEQ consisted of 40 items which aim to explore five main areas that may influence students’ clinical learning. These areas are: provision of clinical cases (6 items), authenticity of clinical experiences (9 items), supervision (7 items), organization of the doctor-patient encounters (11 items), and motivation of students to learn (7 items).

The items of the CLEQ were phrased in a way that could reflect students’ perception. For example: “*I have seen a sufficient number of cases*.” Students respond to each item by rating it on a five point Likert scale as (1) strongly disagree, (2) disagree, (3) undecided, (4) agree and (5) strongly agree. The level of agreement of students is indicated by the mean of their responses to the statements of the CLEQ i.e. the higher the mean, the greater the students’ level of agreement. The details of the items of the first version are shown in the Additional file 1.

### Study context

The medical curriculum in Saudi Arabia is designed in a way that the first two to three years are devoted to the teaching of basic medical sciences. Clinical teaching usually takes place in the following three years. However, new trends in medical education encourage early exposure of medical students to clinical situations [17]. Thus, variation of the timing of clinical training expos ure exists among different medical schools in Saudi Arabia.

The training of undergraduate medical students during clinical rotations is usually organized by rotating them through the main medical specialties: internal medicine, surgery, pediatrics, obstetrics and gynecology and family medicine. Students are also given the option to spend certain time in other disciplines as elective courses.

### Participants

Only undergraduate medical students at the end of their clinical rotations were included in this study, because they were considered to be better able to give their opinion about the different aspects of the clinical learning environment. All students in this phase of their study (N = 182) from three medical colleges in Riyadh City, the capital of Saudi Arabia, were invited to participate. They were instructed that their response, in filling out the questionnaire, should be based on their experience during the clinical rotations and not on their general impression. Students who have less clinical exposure i.e. in the first two years of the curriculum were excluded. One hundred and nine questionnaires were returned (response rate = 60%). The mean age of the participants was 24 years (*sd* = 2.7). In the total sample of the three universities 41% of the participants were females. In the remainder of this article the three medical colleges will be individually referred to as School 1 (50% of the participants), School 2 (24%) and School 3 (26%). The sample of the study has varying level of clinical exposure. Students were either in their 3rd year (14%), 4th year (47%), 5th year (25%) or 6th year (12%); 2% did not answer the question which grade they were in. With respect to rotation the majority of the participants (77%) could be divided over four medical specialties: Pediatrics (28%), Medicine (21%), Family Medicine (16%) and Surgery (12%). Of the remaining 23% of the participants, 17% were in another medical specialty, such as Obstetrics and gynecology, and elective courses, such as, radiology and 6% did not fill in their rotation. The participants in this study are likely to represent the population of undergraduate medical students at these three colleges. This is indicated by the fact that the admission of students to these three medical colleges is based on similar criteria. In addition, the proportion of graduates passing the Saudi licensing examination is similar as well for the three medical colleges.

### Statistical analysis

To investigate the underlying dimensions of the CLEQ a principal component analysis was performed. Since we expected some factors to correlate, as mentioned above, we used Oblimin rotation. Next the reliability of the CLEQ and its dimensions were analyzed computing Cronbach’s alpha. The convergent and divergent validity of the factors represented in the CLEQ was investigated by calculating Pearsons Product Moment correlations between them, therewith testing our hypotheses. Finally, ANOVA’s were used to analyze the mean differences between the three universities on the dimensions of the CLEQ.

The proposal of this study was approved by ethics’ committee of King Abdulla International Research Center (KAIRC), King Saud Ben Abdul-Aziz University for Health Sciences (KSAU-HS), Riyadh, Saudi Arabia.

## Results

### Factor analysis and reliabilities of the factors

First, a factor analysis using principal component analysis with Oblimin rotation was performed, expecting a five-factor structure, because the CLEQ was developed according to the five domains that were expected to influence students’ evaluation of the learning environment. The eigenvalues of the five components were as follows: component 1: 7.7; component 2: 3.0; component 3: 2.5; component 4: 2.2 and finally component 5: 2.0. These five components explained 43% of the total variance. However, a closer inspection of this five-factor structure revealed that a six-factor solution would better fit the data. Four items, now loading on different dimensions, could be better interpreted when allowing them to contribute to another, subsequent dimension. So, next a principal component analysis with Oblimin rotation was performed, expecting a six-factor solution. Of course, the eigenvalues of the first five components were the same as described above; the eigenvalue of the sixth component was 1.8. These six components explained 48% of the total variance. Table 1 shows the factor loadings of all 40 items on these six components together with Cronbach’s alpha and the eigenvalues of these components..

**Table 1:** Oblimin principal component loadings of the items on the six CLEQ-dimension (highest factor loadings in bold)

	**Cases**	**Authenticity**	**Supervision**	**Organization**	**Motivation**	**Self-awareness**
Item 1	**.489**	.225	-.035	-.305	.054	.096
Item 2	**.523**	.285	.088	-.286	.023	.099
Item 3	**.575**	.155	-.215	.152	.055	.157
Item 4	**.664**	-.136	.015	-.084	.162	-.255
Item 5	**.437**	-.319	-.269	-.009	.102	.225
Item 6	**.389**	-.069	.199	-.150	-.007	-.373
Item 7	**.537**	.123	-.120	.303	-.032	.085
Item 8	.213	**.585**	.070	-.186	.025	.191
Item 9	.113	**.537**	-.070	-.213	.031	.191
Item 10	-.008	**.492**	-.186	-.043	.038	-.067
Item 11	**.356**	.080	-.139	-.118	-.260	-.195
Item 12	.003	-.096	-.080	-.056	-.282	-.091
Item 13	.390	.089	.092	-.083	-.336	**-.413**
Item 14	.381	**.512**	.191	.108	-.314	-.163
Item 15	.161	.250	-.141	-.085	-.360	-.302
Item 16	-.021	-.021	**-.711**	-.039	.043	-.057
Item 17	.053	-.225	**-.745**	-.030	.077	-.016
Item 18	-.066	.237	**-.810**	.098	-.006	.134
Item 19	-.010	.022	**-.861**	.043	-.012	.018
Item 20	.100	.098	**-.747**	.064	-.127	.002
Item 21	.126	-.067	.043	-.258	.211	.187
Item 22	-.070	.028	-.378	.136	.091	**-.507**
Item 23	.026	**.455**	-.092	.195	.013	-.325
Item 24	.003	**.646**	-.021	.132	.108	-.017
Item 25	-.117	**.444**	-.067	-.118	.029	-.309
Item 26	.024	**.395**	-.261	-.025	.245	-.267
Item 27	.361	-.070	**-.495**	-.032	.035	-.013
Item 28	.227	-.330	-.288	**-.494**	-.037	-.193
Item 29	-.108	-.113	.093	**-.796**	-.017	-.086
Item 30	.022	.197	.002	**-.675**	-.080	.024
Item 31	-.187	.287	-.292	**-.451**	-.251	-.036
Item 32	-.027	.324	**-.469**	-.316	-.156	.006
Item 33	.066	.258	**-.377**	-.369	-.082	-.182
Item 34	.021	.067	.069	-.080	.115	**-.741**
Item 35	-.074	-.073	-.019	-.042	.094	**-.718**
Item 36	.019	.014	.049	.153	**.775**	-.250
Item 37	.067	.008	-.186	-.024	**.764**	-.221
Item 38	.140	.212	-.069	-.327	**.389**	-.069
Item 39	.111	-.044	-.274	-.164	**.365**	-.194
Item 40	-.026	.257	-.022	-.294	**.362**	-.062
*Alpha*	*.69*	*.75*	*.86*	*.62*	*.70*	*.60*
*Eigenvalue*	*1.8*	*7.7*	*3.0*	*2.5*	*2.2*	*2.0*

It was decided only to interpret items with loadings of .30 or higher because these are probably important and reliable [18]. When an item had two or more factor loadings higher than .30 it was assigned to the factor on which it had the highest loading. Next, we will describe the factors keeping the order of factors as we described them in the introduction, with the added factor Self-awareness as factor 6.

### Description of the six factors

#### Factor 1: Cases (8 items)

Two items that were intended for the factor of Authenticity of clinical experience in the first version of the questionnaire appeared to load higher on the factor of “Cases” than on “Authenticity of clinical experience” and have therefore been added to this factor. These items are item 7: “*I have the opportunity to have the first contact experiences with patients”, and item 11: “I have the opportunity to apply my previous knowledge in patient care”.* These items were closely linked to cases and were considered as features of cases rather than the experience as a whole. Out of the eight items of this factor the statement with the highest loading was: “*I have seen many interesting clinical cases”*. This item was followed by statements with loadings ranging from 0.58 to 0.34, which include statements about the variety and the number of clinical cases. Other items and their loadings are shown in Table 2. The reliability (Cronbach’s α) of this factor was 0.69.

**Table 2:** **Means, standard deviations *, F *-, *p*- and ƞ²-values on the CLEQ-dimensions per medical school (Total n =109)**

**Factor**	**Name of university**	**N**	**M**	**SD**	***F***	***p***	***ƞ*** **²**
Authenticity	School 1	55	2.67	.75	1.27	.28	.01
	School 2	26	2.92	.65			
	School 3	28	2.79	.53			
Supervision	School 1	55	2.88	.83	9.71	.00	.14
	School 2	26	3.51	.71			
	School 3	28	3.54	.63			
Motivation	School 1	55	3.75	.78	.00	.99	.00
	School 2	26	3.75	.68			
	School 3	28	3.76	.53			
Self-awareness	School 1	55	3.11	.81	1.09	.34	.00
	School 2	26	3.11	.65			
	School 3	28	3.35	.78			
Organization	School 1	55	3.11	.64	4.16	.02	.07
	School 2	26	3.54	.67			
	School 3	28	2.99	.96			
Case	School 1	55	3.13	.63	.04	.96	.00
	School 2	26	3.16	.71			
	School 3	28	3.17	.63			

#### Factor 2: Authenticity of clinical experiences (8 items)

The initial version of this factor had nine items (7–15). Five of these items did not emerge in this factor. Two items (7 and 11) had a higher loading on the factor Cases, one item (12) has been deleted because of a non-significant loading, one item (13) had a higher loading on the factor Self-awareness and one item (15) had a higher loading on Motivation. However, four items, expected to belong to the factor of Organization of the patient-doctor encounters, appeared to have the highest loading on this factor. So, finally a total of 8 items loaded high on this factor and the loadings of the items range from 0.65 to 0.40 and Cronbach’s α of this factor was 0.75.

#### Factor 3: Supervision (8 items)

This factor contains eight items and has the highest internal consistency among the other factors (α = 0.86). The 8 items all loaded negatively on this factor. To ease interpretation we multiplied all 8 loadings with -1 [19]. The result of this multiplication is that scoring high on these 8 items means that the participants are satisfied with the received supervision. One statement (item 21) from this factor in the first version of the questionnaire was deleted as it had no significant loading on it. Another item (22) loaded higher on the factor, Self-awareness. In addition, three items (27, 32 and 33) that were intended for the factor Organization of the doctor-patient encounters in the first version of the questionnaire had a higher loading on the factor Supervision and were therefore added to that factor. The highest loading item (0.84) on this factor was *“The way my supervisors deal with medical students was satisfactory”.* This was followed by items related to the commitment of supervisors, their teaching skills, respect of students and their communication skills (0.81, 0.75, 0.74, and 0.71 respectively).

#### Factor 4: Organization of the doctor-patient encounters (4 items)

This factor contained 11 items in the initial version of the questionnaire (items from 23 to 33). However, four items (23, 24, 25 and 26) had higher loadings on the factor Authenticity of clinical experinces, three items (27, 32 and 33) had a higher loading on the factor Supervision. The four remaining items under this factor had loadings ranging from 0.45 to 0.80. The item with the highest loading (.80) was related to the number of students who attend the clinical session, followed by the item about adequacy of time spent with patients (.68). Cronbach’s α for this factor was .62.

#### Factor 5: Motivation to learn (5 items)

This factor contains the expected five items (36–40), however, unexpectedly item 15 also had a high negative loading on this factor. We believe that this item is not well-constructed and leads to confusion among the participants. Therefore, we deleted this item. The highest loading was for the items of: “*I am eager to learn*” (0.78) and “*I am able to look for new information”* (0.76). The reliability was satisfactory: α = .70.

#### Factor 6: Self-awareness (2 items)

Four statements had a significant loading on this factor (13, 22, 34 and 35). Cronbach’s α for this factor was .60.

A total of 3 items were removed from the final questionnaire; items 12 and 21 had no loadings higher than .30 on any of the six factors; item 15 had a high loading on the factor Motivation, but was difficult to interpret, that is, it could not logically be assigned to this factor, probably due to ill-construction of the item. Deleting these three items had a minor positive effect on the internal consistency of the whole questionnaire. Cronbach’s α for all forty items of the questionnaire before removing any item was .87. Taking out item 15 did not have any consequence for the value of Cronbach’s α. Eliminating item 12 and 21 raised Cronbach’s α from .87 to .88. So, Cronbach’s α for the whole questionnaire after removing items 12, 15 and 21 was .88. The next analyses were performed using the mean scores of all participants on the six factors based on the 37 remaining items.

To test the hypotheses that were formulated in the introduction, correlations were computed between the six factors. Table 3 shows these correlations.

**Table 3:** Correlations between the six factors (Total n = 109)

	**1. Cases**	**2. Authenticity**	**3. Supervision**	**4. Organization**	**5. Motivation**	**6. Self-awareness**
**2**	.37**					
**3**	.39**	.41**				
**4**	.31**	.33**	.40**			
**5**	.29**	.26**	.32**	.25*		
**6**	.24*	.36**	.30**	.27**	.30**	

*significant at 0.05 level.

**significant at 0.01 level.

Table 3 shows that all correlations between the six factors are significant at the .01 level with the exception of the correlations between Motivation and Self-awareness and between Self-awareness and Cases, which are significant at the .05 level. These results confirm our expectations mentioned in hypotheses 1 to 5, as far as positive correlations were expected. However, our expectations about the absence of correlations between, for example factor 1 Cases and factor 3 Supervision were not supported. Apparently, all factors relate to each other. This might raise the question whether it is meaningful to differentiate between these six areas of student’s learning evaluation. However, the magnitude of the correlations (all between .24 and .41) indicates that each factor may contribute differently to the quality of the clinical learning environment.

Finally, the mean differences between the three medical schools on the six factors were investigated. Table 2 shows the results.

Table 2 reveals that the three medical schools had significant mean differences on two of the six factors, namely Supervision (*F =* 9.7; *p < .*01), and Organization of the doctor patient encounter (*F =* 4.16; *p < .*02). On the factor Supervision, School 2 and 3 had approximately the same average score (*M =* 3.5) and School 1 had a significantly lower average score (*M =* 2.8). On the factor Organization, School 2 showed the highest mean score (*M =* 3.54); this was significantly different from the mean scores of School 1 (*M =* 3.11) and School 3 (*M =* 2.99). Effect sizes for the mean differences on Supervision and Organization were respectively large and moderate (η^2^ for Supervision = .14 and for Organization = .07). On the other four factors, no significant differences between the three schools were found. Effect sizes ranged from η^2^ = .00 to η^2^ = .01. According to Cohen (1973) values of η^2^ of .01 are considered as a small effect, of .07 as a moderate effect and of .14 and higher as a large effect [20].

## Discussion

In this study, we describe the development of a new instrument (CLEQ) for the evaluation of the clinical learning environment from the perspective of undergraduate medical students. This instrument was needed because of shortcomings in already existing instruments that were developed in the past. The 40 items of the CLEQ were based on our previous study of the perceptions of students and teachers concerning an effective clinical learning environment (8), and on a survey of the literature. These items were placed under five factors, namely Cases, Authenticity of the clinical learning experience, Supervision, Organization of the doctor patient encounter and Motivation to learn. The main aims of the study were to investigate whether these factors could be confirmed by means of factor analysis, and to determine the reliability and validity of the different factors. The study was executed on 182 students coming from three medical schools in Riyadh, Saudi Arabia.

Summarizing, the results have shown that the CLEQ is a multidimensional instrument, which consisted of six factors. In the factor analysis, we found support for the existence of the first five factors mentioned above. However, the data could be better explained by adding a sixth factor, Self-awareness. This last factor has to do with knowing your strengths and limitations as a doctor. The overall internal consistency of the CLEQ is high (Cronbach’s alpha = 0.88). The reliabilities of the six different factors range from reasonable (Cronbach’s alpha = 0.60 for the factor Self-awareness, to 0.86 for the factor Supervision).

After establishing the final factor structure of the CLEQ the items of this instrument were attributed to the factors on which they had the highest loading. Then, to investigate the validity of the different factors, we tested a series of hypotheses concerning the correlations between the factors. Surprisingly, all factors were positively related to each other, therewith giving support to their convergent validity. However, no support was found for their divergent validity. Although, the highly significant correlations between all six factors might indicate that there is no need to differentiate between them, the fact that the correlations are all around .25 -.40 leave room for separate contributions of each single factor to the quality of the learning environment. Moreover, the results have shown that the new instrument is to some extent able to discriminate between the quality of the clinical learning environment of the three different schools that were involved in this study. The scores of the schools differed on the factors Supervision and Organization of the doctor patient encounter.

### Limitations of the study and recommendations

The first limitation of this study is that the CLEQ has been developed and tested in only one country, Saudi Arabia. However, the way in which clinical education is structured has much in common with the way it is structured in other parts of the world. The medical program in one of the schools that was involved in the study is actually based on an Australian medical program. Nevertheless, application of the CLEQ in undergraduate students from medical schools in other countries is needed to evaluate whether we could find comparable psychometric properties as the validity of the instrument could be affected significantly by the characteristics of the involved participants [21,22].

A second limitation is that, for the investigation of the construct validity of the six factors we have not been able to do research into correlations between these factors, that are based upon subjective self-reports of the students, and criteria that have been more objectively determined. So, for example for the factor cases, to have more support for its construct validity, we recommend an investigation in which the scores on this factor in different medical schools with different educational approaches, is correlated with objective data, as logbooks, which helps to gather an objective evidence on how many cases the undergraduate students actually have to deal with. For the factor Supervision the relationship between the subjective scores of the students might be correlated with an objective measure; if, for example, the supervisors are willing to have their supervision sessions video recorded and objectively evaluated by a panel of experts, this might lend more support to the construct validity of the factor Supervision. The construct validities of the more personal factors Motivation and Self-awareness need to be further supported by examining the relationship between the scores of the students on these factors and objectively identified achievements, for example on examinations and assessments of experts on their practical functioning with patients. More information about other dimensions of the validity of the instrument and the magnitude of the influence of each factor on the clinical learning process could be tested further utilizing techniques such as structural equation modeling (SEM) [23].

### Practical use of the CLEQ

The CLEQ is meant to be used in medical undergraduate programs, and results from it can be informative, both for the staff that is responsible for the quality of the clinical rotations and for the students themselves. It offers the opportunity to the staff of these programs to evaluate the quality of their own clinical learning environment. If the scores show that students’ perceptions are that they don’t see enough cases, or not enough difficult cases, or that the authenticity of the clinical learning experiences is questionable, measures have to be taken to improve the system of clinical rotations. If the scores show that students perceive the quality of supervision as too low, supervisors should receive this feedback and try to improve their supervising skills. If the organization of the doctor patient encounter is considered by students as inappropriate, this organization has to be improved. On the other hand, if scores on motivation and self-awareness are low, that might be given as feedback to the students, either on the group level or on the individual level. Of course, in case of high scores on the different clinical environment factors, this might serve as a positive feedback to the people who are responsible for the quality of the program, and high scores on the personal factors motivation and self-awareness can serve as a feedback to the students that they are on the right track to become a good doctor.

## Conclusions

The results of this study support the convergent validity and reliability of the CLEQ. It can be utilized as an evaluation tool for clinical teaching activities, both by educators as well as students. Further research is needed into other dimensions of the validity of the CLEQ. Future confirmatory studies of the validity and quantifying the influence of the variables of the CLEQ in the process of clinical learning are recommended, for example, utilizing techniques such as “structural equation modeling”.

## Competing interests

The authors declare that they have no competing interests.

## Authors’ contributions

All authors contributed to the concept and conduction of this study. AIH initiated the idea and collected the data. AIA, JK, and HTM designed the initial questionnaire, interpreted and analyzed the data and were involved in drafting and revisiting the manuscript. All authors read and approved the final manuscript.

## Pre-publication history

The pre-publication history for this paper can be accessed here:

http://www.biomedcentral.com/1472-6920/14/44/prepub

## Supplementary Material

Additional file 1 Clinical learning evaluation questionnaire (CLEQ) for undergraduate medical education. Click here for file
