# Supplementary material for: Development of the clinical learning evaluation questionnaire for undergraduate clinical education: factor structure, validity, and reliability study
Source: BMC Med Educ. 2014 Mar 4;14:44. doi: 10.1186/1472-6920-14-44 (PMC3944808; doi:10.1186/1472-6920-14-44)
Supplement: Additional file 1 — Clinical learning evaluation questionnaire (CLEQ) for undergraduate medical education. [file 1472-6920-14-44-S1.doc]

**Additional file 1**

**Clinical learning evaluation questionnaire (CLEQ) for undergraduate medical education**

**Cases:**

1. I have seen a sufficient number of clinical cases.
2. I have seen a sufficient number of new clinical cases.
3. I have seen a good variety of clinical cases.
4. I have seen many interesting clinical cases.
5. I have seen some cases with positive clinical findings.
6. I have seen some unusual/rare clinical cases.

**Authenticity of clinical experiences:**

1. I have the opportunity to have the first contact experiences with patients.
2. I am actively involved in the patient care.
3. I have the opportunity to deal with patient as a real doctor.
4. I have the opportunity to deal with the patient as a whole and not limited to a certain system or organ.
5. I have the opportunity to apply my previous knowledge in patient care.
6. I have never been able to write in the patient’s medical chart.
7. I have the opportunity to apply a patient-centered approach.
8. I have the opportunity to take responsibility for patient care.
9. I have the opportunity to communicate with patients and their families.

**Supervision:**

1. My supervisors have good communication skills.
2. I have been respected by my supervisors.
3. The supervisors are committed for teaching.
4. The way my supervisors deal with medical students

is satisfactory.

1. I think supervisors have good teaching skills.
2. I have rarely received a good feedback on my clinical performance from my supervisor.
3. I think that some supervisors could be considered as role models.

**Organization of the doctor-patient encounter:**

1. The objectives of the clinical rotations are clear.
2. Students have some input for the organization and development of the clinical rotations.
3. I have the opportunity to prepare before the clinical encounter.
4. I have the opportunity to reflect and read after the clinical encounter.
5. I have the opportunity to discuss clinical cases with my supervisors.
6. I have the opportunity to share the clinical cases with other students.
7. The number of students in the clinical sessions is appropriate.
8. The time spent with my patients is adequate for my clinical learning.
9. I have the opportunity to utilize skills lab and simulation for clinical cases.
10. I think the assessment of the clinical learning is aligned with objectives.
11. I was given enough assignments during my clinical rotation.

**Motivation\ learning skills:**

1. I adequately know my learning needs
2. I know my limitations.
3. I am eager to learn.
4. I am able to look for new information
5. I come to the clinical sessions prepared and ready.
6. I enjoy learning in clinical sessions.
7. I am able to express myself and show confidence
